# Influence of Silica Nanoparticles on Antioxidant Potential of *Bacillus subtilis* IMV B-7023

**DOI:** 10.1186/s11671-016-1348-2

**Published:** 2016-03-12

**Authors:** Iryna O. Skorochod, Alla O. Roy, Ivan K. Kurdish

**Affiliations:** Department of Microbiological Processes on Solid Surfaces, Zabolotny Institute of Microbiology and Virology, National Academy of Sciences of Ukraine, 154, Acad. Zabolotny St., Kyiv, MSP D03680 Ukraine

**Keywords:** Silica nanoparticles, Antioxidant potential, *Bacillus subtilis*

## Abstract

It was found that if introduced into a nutrient medium of 0.05–1 g/L nano-SiO_2_, the oxidant activity (OA) of the culture medium (CM) of bacilli increased by 43.2–60.1 % and the antioxidant activity (AA) decreased by 4.5–11.8 %. SiO_2_ nanoparticles had different effects on antiradical activity (ARA) of the CM of *Bacillus subtilis* IMV B-7023. In particular, nano-SiO_2_ had no significant effect on the ability of the CM of bacilli to inactivate the 2.2-diphenyl-1-picrylhydrazyl (DPPH·) free radical. However, for the content of the nanomaterial of 0.01–1 g/L decreased hydroxyl radical scavenging in the CM of *B. subtilis* IMV B-7023 on 7.2–17.6 % compared with a control. Low doses of silica nanoparticles stimulated the reducing power of the CM of bacteria and then highly suppressed it.

## Background

High antioxidant and antiradical properties of *Bacillus subtilis* IMV B-7023 [1] allow the recommend of bacterial preparations that are based on this strain for crops which are exposed to aggressive stress agents. Note, however, that the introduction of these organisms into agroecosystem will have an influence on disperse materials of various nature [2], in particular nanomaterials, the dimensions of which are at least in one geometric dimension of less than 100 nm [3]. In nanocondition, substances acquire a number of new physical and chemical characteristics that differ significantly from the original in the same substances of micron size or larger size [4].

The unique properties of nano-sized silica, such as high specific surface area, mechanical and thermal resistance, the ability to pass UV radiation, and the lack of photodegradation, found their application in various fields [5]. However, some authors [5–7] indicate that nano-SiO_2_ inherent the oxidative effect in living organisms. Accordingly, the purpose of this work was to study the influence of silica nanoparticles on antioxidant and antiradical properties of *B. subtilis* IMV В-7023.

## Methods

### Microorganisms, Nutrient Media, and Culture Conditions

The phosphate-mobilizing bacteria *B. subtilis* IMV B-7023 [8] were isolated at the Department of Microbiological Processes on Solid Surfaces, Zabolotny Institute of Microbiology and Virology, National Academy of Sciences of Ukraine. The strain *B. subtilis* IMV B-7023 was grown in 750 mL Erlenmeyer flasks with 100 mL of the Spizizen glucose-mineral liquid medium (g/L): (NH_4_)_2_SO_4_ 2.0, K_2_HPO_4_·3H_2_O 14.0, KH_2_PO_4_ 6.0, trisodium citrate dihydrate 1.0, MgSO_4_·7H_2_O 0.2, and glucose 10.0 (рH 7.0–7.2) [9]. The initial bacterial concentration after inoculation was 10^6^ cells/mL. Incubation was performed under batch conditions at 28 °C with shaking at 240 rpm for 22 h. Then, studies were carried out in the “acute experiment” that allowed to evaluate the response of the antioxidant system of *B. subtilis* IMV B-7023 to make the nutrient medium of the nanomaterial. The suspension of bacilli was received in a number of flasks containing more than 10^8^ cells/mL, averaged and added on 100 mL flasks with sterile weighed quantities of nano-SiO_2_ (0.01–1.00 g/L), and cultivated during 2 h in the conditions described above. In the control, the bacteria were cultivated in a nutrient medium without the nanomaterial.

The culture liquid of *B. subtilis* IMV B-7023 after completion of their growth was freed from the cells of bacteria and nano-SiO_2_ by centrifugation on the centrifuge OPn-8 (joint stock company “TNK DASTAN,” Kirgizstan) during 25 min at 5000*g*. In the obtained culture medium (CM) of *B. subtilis* IMV B-7023, the indices of antioxidant potential were determined.

### Nanomaterial

Nano-sized silica was kindly provided by Chuiko Institute of Surface Chemistry, National Academy of Sciences of Ukraine. The size of the silica nanoparticles was 5–20 nm [10].

### Assay of Antioxidant Activity

The antioxidant activity (AA) level in the CM of *B. subtilis* IMV B-7023 was estimated by measuring the thiobarbituric acid reactive substances (TBARS) following Tween 80 oxidation. This level was determined spectrophotometrically at 532 nm [11, 12]. The assay of TBARS measures malondialdehyde (MDA) present in the sample as well as MDA generated from lipid hydroperoxides by the hydrolytic conditions of the reaction. The CM of *B. subtilis* IMV B-7023 inhibits the Fe^2+^/ascorbate-induced oxidation of Tween 80, resulting in a decrease in the TBARS level. Briefly, 1.0 mL of the CM of bacilli was added to 2.0 mL of 1 % Tween 80 reagent, 0.2 mL of 1 Mm FeSO_4_, and 0.2 mL of 10 Mm ascorbic acid. In the control assay, 1 mL of nutrient media was used instead of the sample. The mixture was heated in a boiling water bath for 48 h at 40 °C. After cooling, 1.0 mL of 40 % trichloroacetic acid (TCA) was added. After 60 min, the mixture was centrifuged at 5000*g* for 15 min. After centrifugation, 1.0 mL of supernatant and 2.0 mL of 0.25 % of thiobarbituric acid (TBA) reagent were mixed. The mixture was heated in a boiling water bath at 95 °C for 15 min. The absorbance of the obtained solution was measured at 532 nm using a UV-46 spectrophotometer (joint stock company “Leningrad Optical-Mechanical Association (LOMO),” Russia). The level of AA in the sample (%) was calculated using the following equation:

1 $$ \mathrm{AA}=\frac{A_{\mathrm{control}}-{A}_{\mathrm{sample}}}{A_{\mathrm{control}}}\cdot 100\ \% $$

where *A*_sample_ is the absorbance in the presence of the sample of the CM of *B. subtilis* IMV B-7023 and *A*_control_ is the absorbance of the control. The control contains all reagents except the CM of *B. subtilis* IMV B-7023. All tests were performed in triplicate, and the mean was centered.

### Assay of Oxidant Activity

Oxidant activity (OA) of the CM of *B. subtilis* IMV B-7023 was assessed by the accumulation in a model system, the end product of lipid peroxidation (LPO) such as MDA [12]. The substrate used was Tween 80, and the initiator of LPO was the CM of bacilli. Briefly, 1.0 mL of the CM of *B. subtilis* IMV B-7023 was added to 2.0 mL of 1 % Tween 80 reagent. In the control assay, 1 mL of nutrient media was used instead of the sample. The mixture was heated in a boiling water bath for 48 h at 40 °C. After cooling, 1.0 mL of 40 % TCA was added. After 60 min, the mixture was centrifuged at 5000*g* for 15 min. After centrifugation, 2.0 mL of supernatant and 2.0 mL of 0.25 % TBA reagent were mixed. The mixture was heated in a boiling water bath at 95 °C for 15 min. As a result of the reaction, two molecules of TBA with one molecule of MDA produce a trimethine complex having a pink color. The absorbance of the obtained solution was measured at 532 nm using a UV-46 spectrophotometer (joint stock company LOMO, Russia). The level of OA in the sample (%) was calculated using the following equation:

2 $$ \mathrm{O}\mathrm{A}=\frac{A_{\mathrm{sample}}-{A}_{\mathrm{control}}}{A_{\mathrm{sample}}}\cdot 100\ \% $$

where *A*_sample_ is the absorbance in the presence of the sample of the CM of *B. subtilis* IMV B-7023 and *A*_control_ is the absorbance of the control. The control contains all reagents except the CM of *B. subtilis* IMV B-7023. All tests were performed in triplicate, and the mean was centered.

### Reducing Power Assay

The reducing power of the CM of *B. subtilis* IMV B-7023 was analyzed according to the method of Oyaizu [13]. The ability of the CM of bacilli to reduce the K_3_[Fe^3+^(CN)_6_] to K_4_[Fe^2+^(CN)_6_] was determined by recording the absorbance at 700 nm after incubation. For this purpose, 1.0 mL of the CM of the studied strain of bacilli was mixed with phosphate buffer (2.5 mL, 0.2 M, pH 6.6) and potassium ferricyanide (K_3_[Fe^3+^(CN)_6_]) (2.5 mL, 1 %). The mixture was incubated at 50 °C for 20 min. A portion (2.5 mL) of 10 % TCA was added to the mixture, which was then centrifuged (1000*g* at room temperature) for 10 min. The upper layer of the solution (2.5 mL) was mixed with distilled water (2.5 mL) and FeCl_3_ (0.5 mL, 0.1 %), and the absorbance was measured at 700 nm using a UV-46 spectrophotometer (joint stock company LOMO, Russia). Increased absorbance of the reaction mixture indicated increased reducing power. All tests were performed in triplicate, and the mean was centered.

### DPPH· Radical Scavenging Activity

The free radical scavenging activity of the CM of *B. subtilis* IMV B-7023, based on the scavenging activity of the stable 2.2-diphenyl-1-picrylhydrazyl (DPPH·) free radical, was determined by the method described by Shimada et al. [14]. The rapid reaction between antioxidants (AH) and DPPH· occurs with the transfer of the most labile H atoms to the radical, while the subsequent slow step depends on the residual H-donating capacity of antioxidant degradation products [15]: DPPH·+AH→DPPH·−H+A·. AH reacts with DPPH·, which is a stable free radical, and converts it to a stable diamagnetic molecule (2.2-diphenyl-1-picrylhydrazine). Briefly, 0.1 mM solution of DPPH· in ethanol was prepared and 1 mL of this solution was added to 3.0 mL of the CM of bacilli. The mixture was shaken vigorously and allowed to stand at room temperature for 30 min. The control was added with 3.0 mL of a nutrient medium. Then, the absorbance was measured at 517 nm using a UV-46 spectrophotometer (joint stock company LOMO, Russia). Lower absorbance of the reaction mixture indicated higher free radical scavenging activity. The percent DPPH· scavenging effect was calculated using the following equation:

3 $$ \mathrm{DPPH}\cdot \kern0.28em \mathrm{scavenging}\kern.5em \mathrm{effect}\kern0.75em \left(\%\right)=\left[1-\left(\frac{A_{\mathrm{sample}}}{A_{\mathrm{control}}}\right)\right]\cdot 100\ \% $$

where *A*_sample_ is the absorbance in the presence of the sample of the CM of *B. subtilis* IMV B-7023 and *A*_control_ is the absorbance of the control. The control contains all reagents except the CM of *B. subtilis* IMV B-7023. All tests were performed in triplicate, and the mean was centered.

### Hydroxyl Radical Scavenging Assay

The scavenging ability of the CM of *B. subtilis* IMV B-7023 on hydroxyl radicals was determined according to the method described by Smirnoff and Cumbes [16] with some modifications [17]. Briefly, the individual sample of the CM of bacilli (3.0 mL) was added to the reagent containing 1.0 mL of 1.5 mM FeSO_4_, 0.7 mL of 6 mM H_2_O_2_, and 0.3 mL of 20 mM sodium salicylate. The control was added with 3.0 mL of a nutrient medium. After incubation for 1 h at 37 °C, the absorbance of the hydroxylated salicylate complex was measured at 562 nm using a UV-46 spectrophotometer (joint stock company LOMO, Russia). The scavenging ability on hydroxyl radicals was calculated using the following equation:

4 $$ \mathrm{Scavenging}\ \mathrm{ability}\ \mathrm{on}\ \mathrm{hydroxyl}\ \mathrm{radicals}\kern0.5em \left(\%\right)=\left[\frac{\left({A}_{\mathrm{control}}-{A}_{\mathrm{sample}}\right)}{{\mathrm{A}}_{\mathrm{control}}}\right]\cdot 100\ \% $$

where *A*_control_ is the absorbance of the control reaction (containing all reagents except the samples of the CM of bacilli) and *A*_sample_ is the absorbance in the presence of the sample of the CM of *B. subtilis* IMV B-7023. All tests were performed in triplicate, and the mean was centered.

### Statistical Analysis

Microsoft Excel (Microsoft Corporation, USA) was used to analyze the data on the average of the three replicates (±SE) obtained from the three independent experiments. Differences were compared with the statistical significance at a *P* level less than 0.05 (*P* < 0.05). The Kolmogorov-Smirnov test was used to assess the normality of the distribution of each treatment [18].

## Results and Discussion

Silica nanoparticles can easily penetrate into the cells [19], but increasingly, their biological effect is associated with the pronounced membranotropic properties. Underlying of these properties are electrostatic attraction and formation of the hydrogen bond between the silanol groups on the surface of silica nanoparticles and active centers of membrane phospholipids and proteins [20]. According to the literature [5, 7], silica nanoparticles interact with the lipid bilayer of cell membranes that can stimulate the excessive formation of reactive oxygen species (ROS), which are biological factors of the peroxidation of bio-effecting molecules [6].

**Fig. 1:**
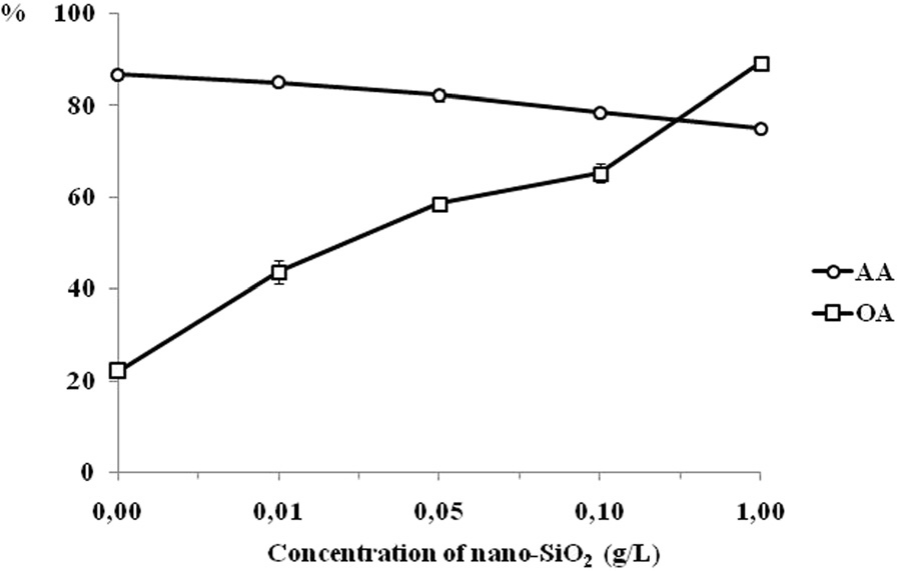
Effect of different doses of silica nanoparticles on antioxidant activity (*AA*) and oxidant activity (*OA*) of the culture medium of *Bacillus subtilis* IMV B-7023

In studying the effect of different doses of nano-SiO_2_ on antioxidant potential of *B. subtilis* IMV B-7023, it was established that this nanomaterial is characterized by a pronounced prooxidant effect. According to the research of oxidant and antioxidant activities of the CM of bacilli, it was shown that by culturing the bacteria with 0.01 g/L of nanodispersed SiO_2_, no significant changes were observed in the AA. However, OA increased by 21.7 % compared with a control (Fig. 1). With increasing doses of the nanomaterial from 0.05 to 1 g/L, AA decreased by 4.5–11.8 % and OA increased by 43.2–60.1 % (Fig. 1).

**Fig. 2:**
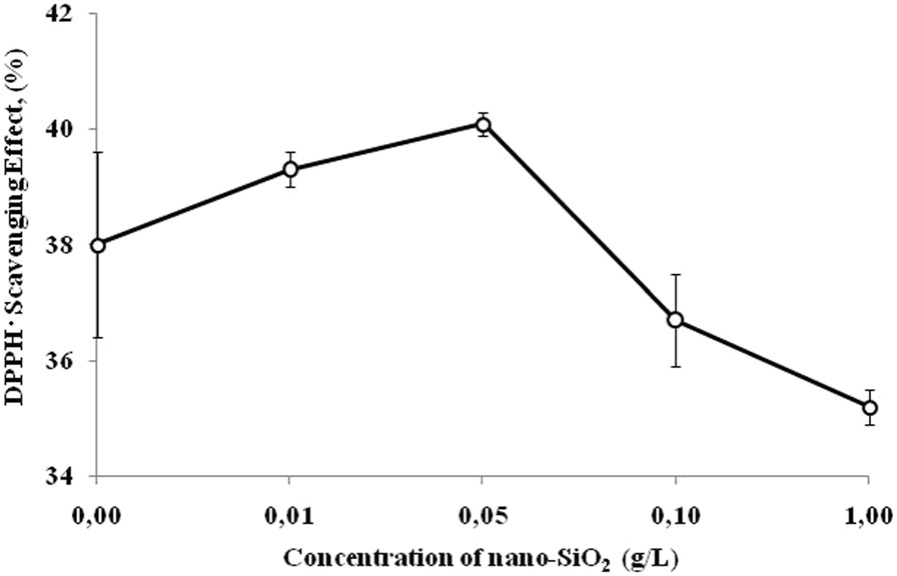
Antiradical activity of the culture medium of *Bacillus subtilis* IMV B-7023 for the different contents of silica nanoparticles in the nutrient medium

We have shown that silica nanoparticles cause a different effect on antiradical activity (ARA) of the CM of *B. subtilis* IMV B-7023 towards DPPH· and ·OH. In particular, nanodispersed SiO_2_ had no significant effect on the ability of the CM of bacilli to inactivate the DPPH·. Thus, at culturing bacilli with 0.01–0.05 g/L of nano-SiO_2_, ARA increased by 1.3–2.1 %. When the content of the nanomaterial in the nutrient medium was 1 g/L, the investigational indicator decreased by 2.8 % compared with the control (Fig. 2). It should be assumed that the indicators of ARA of the CM of *B. subtilis* IMV B-7023 remained at a high level regardless of the introduced dose of nano-SiO_2_ by virtue of the ability of these bacteria to produce phenolic compounds [21]. These compounds, according to published data, may have inherent pronounced antiradical properties [22, 23].

**Fig. 3:**
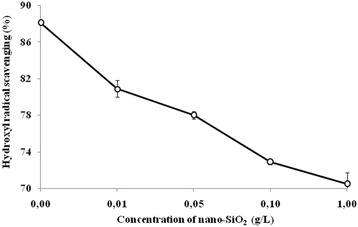
The impact of the silica nanoparticles on the inhibition in the culture medium of *Bacillus subtilis* IMV B-7023. The hydroxyl radical is a product of Fenton’s reaction

However, the silica nanoparticles inhibited the hydroxyl radical (·OH) scavenging in the CM of *B. subtilis* IMV B-7023. It was found that, if introduced into the nutrient medium of 0.01–0.05 g/L of nano-SiO_2_, the investigated parameter was below the control by 7.2–10.1 %. By increasing the content of the nanomaterial to 1 g/L, the hydroxyl radical scavenging in the CM of bacilli decreased relative to the control at 17.6 % (Fig. 3).

No detailed mechanism of accumulation of oxidants in living cells with the participation of various nanomaterials was found out. According to the published data [24–26], the surface of nano-sized silica particles in an aqueous medium can be generated hydrogen peroxide, singlet oxygen, hydroxyl radical, and other ROS.

Shi et al. [27] and Lingard et al. [28] showed that the concentration of ·OH is closely correlated with the size of nanoparticles; the smaller the particle of nano-SiO_2_, the more this radical is formed. According to the results of Yu et al. [29], the hydroxyl-generating activity of nanodispersed silica depends not only on the size of its particles but also on the content of adsorbed iron ions on the surface of the nanomaterial. It was established that the addition of H_2_O_2_ to Fe^3+^-containing nano-SiO_2_ causes the excessive formation of ·OH for the mechanism of Fenton’s reaction, which occurs on the surface of particles of the nanomaterial [30]. Some scientists also believe that the relatively high content of metal ions in nanomaterials can play a key role in the formation of hydroxyl radical by Fenton’s reaction [31].

In our studies, we used nano-sized silica, the purity of which was not less than 99.9 %, and the mass fraction of Fe^3+^-containing impurities amounted to only 0.002 % [32]. Fenoglio with co-authors [33, 34] found that SiO_2_ nanoparticles can generate hydroxyl radical in the absence of the adsorbed iron ions on their surface. However, according to the literature [35], the mechanism of the formation of ·OH could play an active role in superoxide anion radical (O_2_^−·^), which is also generated on the surface of nano-SiO_2_ in an aqueous medium. O_2_^−·^ acts as a reductant of metal ions or reaction sites on the surface of nano-sized silica. Redox reactions that occur with the participation of the oxidant can contribute to nano-SiO_2_-mediated accumulation of ·OH:

1. О_2_^− ·^ + М^*n* +^ → M^(*n* − 1)^ + O_2_;
2. M^(*n* − 1)^ + H_2_O_2_ → M^*n* +^ + ОН + OH^−^;
  M^*n*+^ / M^(*n* − 1)^
3. О_2_^− ·^ + H_2_O_2_ → ОН + O_2_ + OH^−^,

where M^*n*+^ are the metal ions or the reaction sites on the surface of nano-SiO_2_. Reactions 1–3 are reactions of type Haber-Weiss [24, 35]. The hydroxyl radical, which formed in the course of these reactions, can be site-specifically generated on the surface of nano-SiO_2_ and can effectively attack DNA [35].

**Fig. 4:**
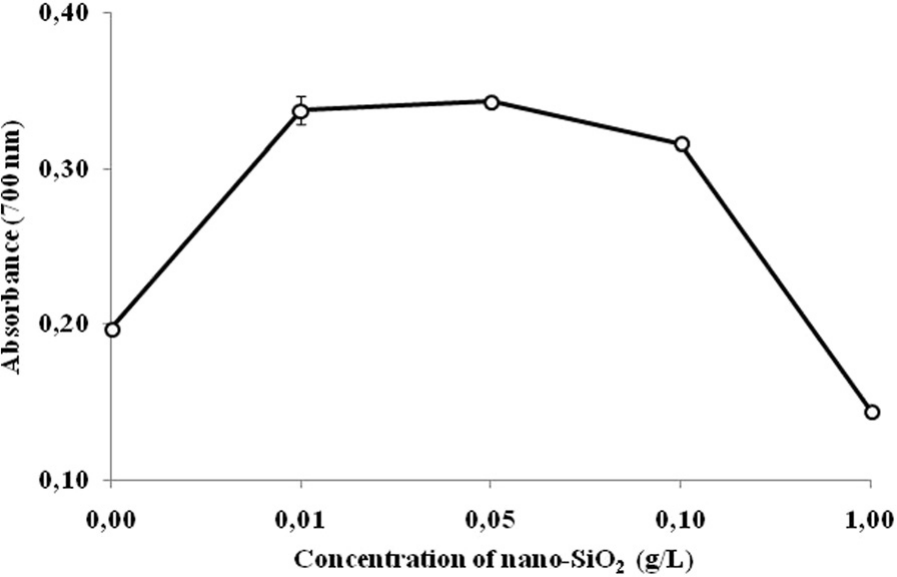
The effect of different doses of silica nanoparticles on the reducing power of the culture medium of *Bacillus subtilis* IMV B-7023. The absorbance of the product of reaction between FeCl_3_ and K_4_[Fe(CN)_6_] was used as indicator

The silica nanoparticles had a noticeable influence on the reducing power of the CM of *B. subtilis* IMV B-7023. So, absorption to the control variant amounted to 0.197. By culturing bacteria with 0.01–0.05 g/L of nanodispersed SiO_2_, the investigated index increased in comparison with the control and amounted to 0.337–0.343. With increasing doses of the nanomaterial up to 1 g/L, a sharp decline of the reducing power of the CM of *B. subtilis* IMV B-7023 was observed to be 0.144 (Fig. 4). This effect of nano-SiO_2_ on the reducing power of the COP of the investigated strain of bacteria may be associated with the increased content of ROS [24, 26].

## Conclusions

Thus, low concentrations of silica nanoparticles caused a moderate prooxidant effect on the background of activation of antioxidant defense factors of *B. subtilis* IMV В-7023. However, high doses of the nanomaterial suppressed a number of indicators of the antioxidant potential of the studied strain of the bacilli. The mechanism by which nano-sized silica generates ROS requires further study.

## Abbreviations

AA: antioxidant activity
AH: antioxidants
ARA: antiradical activity
CM: culture medium
DPPH·: 2.2-diphenyl-1-picrylhydrazyl
LPO: lipid peroxidation
MDA: malondialdehyde
OA: oxidant activity
ROS: reactive oxygen species
TBA: thiobarbituric acid
TBARS: thiobarbituric acid reactive substances
TCA: trichloroacetic acid
O_2_^−·^: superoxide anion radical
·ОН: hydroxyl radical
